# Primary cilia-associated signalling in squamous cell carcinoma of head and neck region

**DOI:** 10.3389/fonc.2024.1413255

**Published:** 2024-08-21

**Authors:** Iveta Putnová, Barbora Moldovan Putnová, Pavel Hurník, Jan Štembírek, Marcela Buchtová, Petra Kolísková

**Affiliations:** ^1^ Laboratory of Molecular Morphogenesis, Institute of Animal Physiology and Genetics, Czech Academy of Sciences, Brno, Czechia; ^2^ Department of Anatomy, Histology and Embryology, University of Veterinary Sciences Brno, Brno, Czechia; ^3^ Department of Pathological Morphology and Parasitology, University of Veterinary Sciences Brno, Brno, Czechia; ^4^ Institute of Molecular and Clinical Pathology and Medical Genetics, University Hospital Ostrava, Ostrava, Czechia; ^5^ Institute of Molecular and Clinical Pathology and Medical Genetics, Faculty of Medicine, University of Ostrava, Ostrava, Czechia; ^6^ Department of Maxillofacial Surgery, University Hospital Ostrava, Ostrava, Czechia; ^7^ Department of Experimental Biology, Faculty of Science, Masaryk University, Brno, Czechia

**Keywords:** head and neck cancer, primary cilium, Hedgehog, Wnt, PDGF, oral squamous cell carcinoma, signalling pathway inhibitors

## Abstract

Squamous cell carcinoma (SCC) of the head and neck originates from the mucosal lining of the upper aerodigestive tract, including the lip, tongue, nasopharynx, oropharynx, larynx and hypopharynx. In this review, we summarise what is currently known about the potential function of primary cilia in the pathogenesis of this disease. As primary cilia represent a key cellular structure for signal transduction and are related to cell proliferation, an understanding of their role in carcinogenesis is necessary for the design of new treatment approaches. Here, we introduce cilia-related signalling in head and neck squamous cell carcinoma (HNSCC) and its possible association with HNSCC tumorigenesis. From this point of view, PDGF, EGF, Wnt and Hh signalling are discussed as all these pathways were found to be dysregulated in HNSCC. Moreover, we review the clinical potential of small molecules affecting primary cilia signalling to target squamous cell carcinoma of the head and neck area.

## 1 Squamous cell carcinoma in the head and neck area and its main characteristics

Head and neck tumours represent the seventh most common cancer type worldwide. Approximately 900,000 new cases are diagnosed each year, with head and neck squamous cell carcinoma (HNSCC) accounting for more than 90% of all oral malignant cases (1). The incidence and mortality rates vary with gender, where a generally higher occurrence is found in males compared to females in a ratio of 2:1 in most countries (2). Despite improvements in diagnosis, surgical techniques and various strategies for treatment, the 5-year survival rates of HNSCC throughout the world have not improved. The most common reasons for this include distant metastases, the progression of second primary malignancies, cancer recurrence and resistance to chemotherapy and radiation therapy (3–5).

The mechanisms contributing to the initiation of head and neck carcinomas are very complex, as is their classification. These tumours usually originate in the squamous cells, which cover the majority of the mucosal surfaces in the head and neck region, and together they are referred to as head and neck squamous cell carcinoma (6). Despite their common origin in the squamous mucosa of the upper aerodigestive tract, HNSCC exhibits wide heterogeneity, which is based on several sources. This fact complicates their consideration as a single disease entity. The head and neck regions are made up of multiple distinct structures in terms of morphology (e.g., lip, tongue, mouth floor, palate, jaw, nasopharynx, oropharynx, larynx and hypopharynx) with typical microscopic features, lymphatic and vascular drainage or innervation (7). Thus, there are several types of HNSCC, including oral squamous cell carcinoma (OSCC), oropharyngeal squamous cell carcinomas (OPSCC), laryngeal squamous cell carcinoma (LSCC) and hypopharyngeal squamous cell carcinoma (HSCC) (8). OSCC, which makes up over half of HNSCC, develops in the squamous epithelium of the lip or oral cavity lining (9). Due to their frequent detection at stages III and IV (locally progressed), most OSCC (60%) have a poor prognosis, with just 30% of cases surviving for five years (10).

Different HNSCC types exhibit distinct molecular characteristics associated with variable clinical progression, available treatment approaches and their outcomes (11). Alcohol use, tobacco smoking and human papillomavirus (HPV) infection are among the risk factors for HNSCC (12). However, young patients without any known risk factors might develop HNSCC (7), as was proven in a study on non-smoking, non-drinking, HPV-negative patients with OSCC, who were characteristic by the occurrence of various molecular alterations, in most cases (60%), a mutation in gene *TP53* (coding tumour suppressor protein p53) (13).

Currently, the standard treatment option represents radical surgical resection of tumours in combination with chemotherapy and radiation, which provides limited efficacy in advanced cases (12). Targeted therapies exist as an alternative to conventional chemotherapies, which are harmful to both healthy and tumour cells. They are precisely designed to prevent the function of certain signalling proteins, the activity of which is mainly limited to malignant tissue. In targeted therapy, antibodies or small molecule inhibitors, which selectively block signal transduction pathways linked to growth, proliferation and survival, may be used (14). Given the success of molecularly targeted drugs in treating various malignancies, it is encouraging to develop new treatment options for HNSCC based on molecular changes observed in these patients (15). Here, we focus on signalling associated with primary cilia, as it represents a promising target, opening new avenues for treatment.

## 2 Introduction to primary cilia

**Figure 1:**
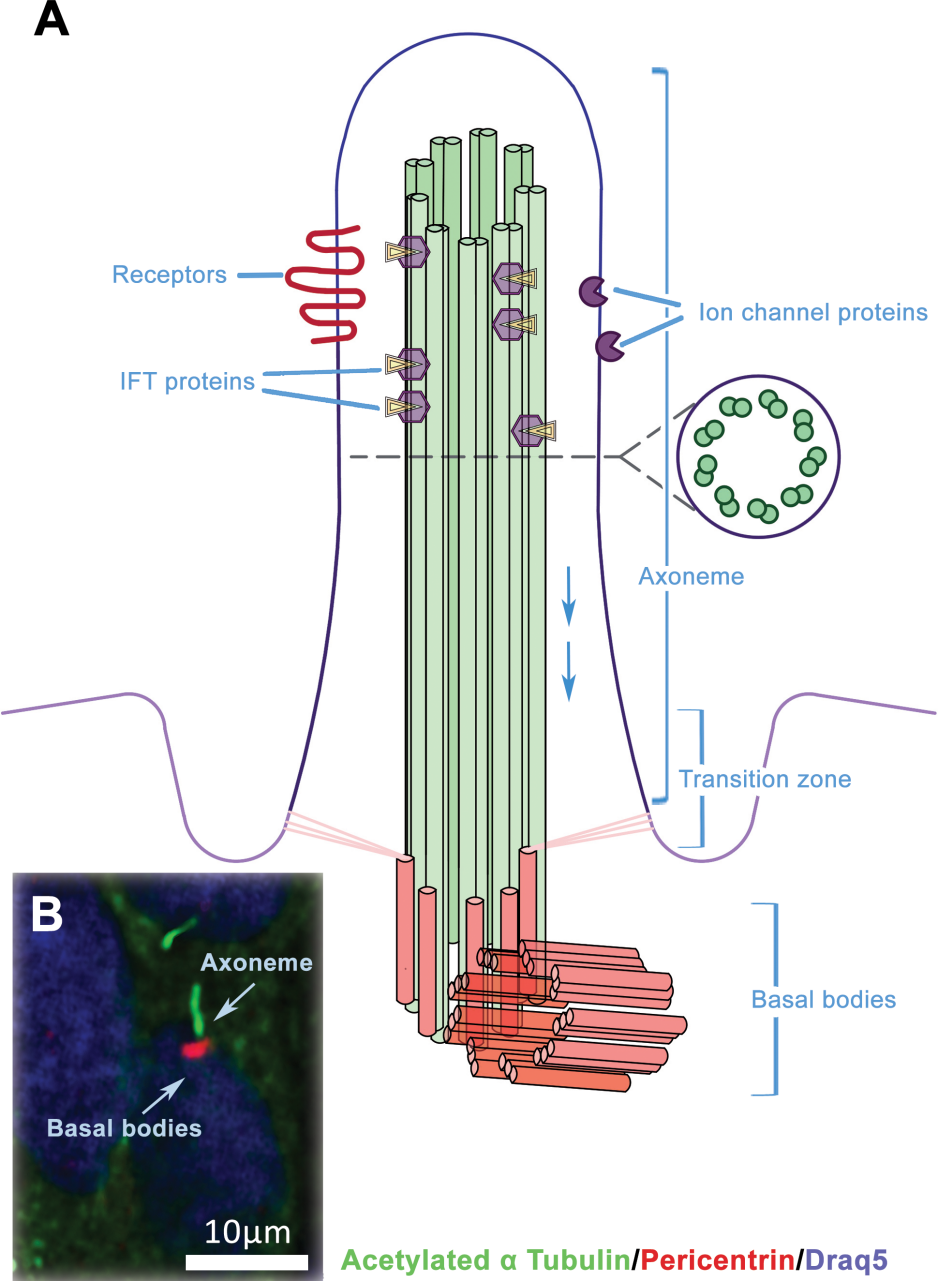
The structure of primary cilium. **(A)** The primary cilium consists of axonemal microtubules, basal body and plasmatic membrane. The ciliary membrane is different from the membrane, which surrounds the rest of the cell and includes various receptors and transmembrane ion channels. Intraflagellar transport (IFT) is active along the axonemal microtubules as bidirectional movement of protein particles, and it is necessary for the assembly and maintenance of primary cilium. **(B)** Example of immunofluorescent visualisation of the primary cilia – the axonema (green) and basal body (red), chromatin in the nucleus is counterstained by DRAQ5 (blue).

Primary cilia (PC) are microtubule-based organelles that are formed from a centriolar basal body (**Figure 1**). These structures, which are made up of more than 600 proteins, extend from the apical surface of the majority of mammalian cells and have been remarkably conserved during evolution (16). Their appearance on the cell surface is unique to specific stages of the cell cycle (17). The presence of the primary cilium and cell proliferation are inversely correlated in vertebrates. During cell division, the cilium is reabsorbed into the cell, allowing the centrosome to function in the mitotic apparatus (18). In contrast to the well-known motile cilia (such as in the ciliated epithelium of mucous membranes that line human airways, sperm flagella etc.), primary cilia are generally non-motile (except for nodal cilia) and located as a single cilium per cell (19). Axonemes in non-motile primary cilia contain 9 + 0 microtubules, and this same structure can be found on epithelial cells (e.g. kidney tubules) as well as non-epithelial cells such as fibroblasts and neurons (20). The transfer of molecules along the axoneme is mediated by intraflagellar transport (IFT). Two distinct microtubule motors move IFT particles along the microtubule: cytoplasmic dynein 2 moves retrogradely (towards the cell body), and heterotrimeric kinesin-2 moves anterogradely (towards the cilia tip) (21, 22). The transition zone, located between the cilium and basal body, contains unique gating structures that, in conjunction with the transition fibres in the basal body, regulate the entry and exit of ciliary proteins. This mechanism helps to compartmentalise the organelle (23).

The primary cilia function as a cellular ‘antenna’ that receives diverse signals from the extracellular environment, including those related to fluids, odours and light. The primary cilium is also an important signal mediator because it exhibits both the protein receptors needed for signal interception and the downstream molecular effectors. To date, the association of primary cilia with Hedgehog, Wnt and platelet-derived growth factor signalling is the best characterised (24, 25). A number of receptor tyrosine kinases, including epidermal growth factor receptor (26, 27), fibroblast growth factor receptor 3 (28), insulin-like growth factor receptor (29), angiopoietin-1 receptor (30) and transforming growth factor beta receptor (31, 32), were also found to localise to primary cilia and affect their function.

### 2.1 Primary cilia-associated signalling

#### 2.1.1 Platelet-derived growth factor signalling

Numerous cell types, including neurons, corneal epithelial cells, fibroblasts, endothelial cells, smooth muscle cells, macrophages and preosteoclasts, express platelet-derived growth factor (PDGF), originally identified in platelets (33). PDGFR signalling belongs to the receptor tyrosine kinase pathways and depends on two receptors (PDGFRα and PDGFRβ) and four ligands (PDGF A-D), which can form five subtypes of PDGF dimers. Binding of the ligand (dimer) causes homo- or hetero-dimerisation of the receptor and subsequent autophosphorylation on the tyrosine residues of the receptors, which serves as a platform for the binding of downstream molecules. Upon binding to their corresponding PDGFRs, PDGF isoforms trigger the dimerisation and activation of distinct receptors, hence offering several binding sites for numerous signalling molecules. This makes it possible to activate a number of signalling pathways, including the Notch, phosphatidylinositol 3 kinase (PI3K)/protein kinase B (AKT/PKB), mitogen-activated protein kinase (MAPK)/extracellular signal-regulated kinase (ERK) and Janus kinase (JAK)/signal transducers and activators of transcription (STAT) pathways (24, 34). PDGFRα was found to be restricted to the primary cilia in growth-arrested fibroblasts (35) or in the neural stem cells of the adult rat subventricular zone (27). Moreover, ciliary localisation was detected in the Mek1/2 downstream signalling cascade components (24). Furthermore, fibroblasts originating from Tg737orpk mutants (Tg737 encodes the IFT particle protein IFT88/Polaris necessary for ciliary assembly) cannot generate typical cilia and exhibit an increase in PDGFRα expression (35).

#### 2.1.2 Wnt signalling

Primary cilia have also been associated with Wnt signalling, categorised as either β-catenin dependent (the so-called canonical pathway) or β-catenin independent (the noncanonical pathways); however, there are still discussions about their direct or indirect role in signalling. The canonical pathway is initiated by binding of Wnt ligands to Frizzled (FZD) receptors and the lipoprotein-receptor related protein (LRP) co-receptors (LRP5/6), which activates the cytoplasmic phosphoprotein Dishevelled (DSH/DVL). This results in the inhibition of the destruction complex that constantly degrades β-catenin in the cytoplasm. Thus, stabilised β-catenin can translocate to the nucleus, where it interacts with other transcription factors and triggers gene expression related to cell proliferation, differentiation and metabolism (36). Several members of the Wnt signalling pathway have been identified to localise to the cilium, including FZD3, DSH2, β-catenin and glycogen synthase kinase-3β (GSK3β) (37) or in proximity to the cilium, such as VANGL-2 and APC (38). Mouse embryos lacking ciliary proteins have aberrant β-catenin and impaired canonical Wnt responses (39). Moreover, primary cilia-associated proteins (such as Kinesin-2 and IFT-A proteins) have been determined to be necessary for the fine tuning of Wnt signalling (40). Next, the elevation of β-catenin levels was shown to be related to the reduction of ciliogenesis (41, 42) and primary cilium disassembly to trigger an abnormal Wnt signalling pathway (43, 44). Inversin, a protein identified at the primary cilium, may interact with DVL and influence its degradation, mediating the transition between canonical and noncanonical Wnt signalling (45). Noncanonical Wnt pathways can be further distinguished into the Planar Cell Polarity (PCP) pathway and the Wnt/Ca^2+^ pathway, which does not require β-catenin and likewise begins with a Wnt ligand binding to the FZD receptor. Both pathways exhibit diverse roles during embryogenesis, where PCP is crucial in the regulation of the actin cytoskeleton for polarised organisation of epithelial structures or directed migration (46), and a link between PCP signalling and the cilium has been demonstrated in several studies (47, 48).

#### 2.1.3 Hedgehog signalling

Hedgehog (Hh) signalling, the best studied cilia-related signalling pathway, is crucial for vertebrate embryonic development and also plays a role in the adult tissues, including cytoskeletal rearrangement, cell migration, proliferation and cellular maintenance (49). Of the three mammalian Hh ligands, Sonic hedgehog (SHH), Desert hedgehog (DHH) and Indian hedgehog (IHH), SHH is the best studied. Briefly, the Hh pathway is activated by binding of the ligand to its transmembrane receptor Patched (PTCH). In the absence of Hh signals, PTCH1 keeps the transmembrane protein Smoothened (SMO) inactive. When PTCH1 binds to a ligand, PTCH1 loses the ability to suppress SMO, leading to the activation of GLI transcriptional factors, which are pathway effectors (50). There are three GLI proteins (GLI1, GLI2 and GLI3), and their post-translational modifications result in the intracellular activation or repression of pathway targets in the presence or absence of Hh ligands, respectively, to establish the ultimate transcriptional output of canonical Hh signalling (51).

From a ciliary point of view, SMO is kept out of the cilium in the absence of Hh ligand while PTCH1 is localised to the ciliary membrane (52, 53). GLI transcription factors are converted into the repressor form, which prevents Hh proteins from being transcriptionally activated (51). When Hh is present, SMO is phosphorylated and translocated into the cilium, where it stimulates GLI activation, while receptor PTCH1 moves out of the cilium (52, 54). GLI in the activator form is transported into the nucleus where it activates the transcription of Hh target genes (51). The protein Suppressor of Fused (SUFU), which is involved in the production of both the repressor and activator versions of GLI proteins, is another means of cilia-dependent control of GLI proteins (55). SUFU sequesters GLI protein to the cytoplasm and maintains it in the repressor form when HH is absent. When HH is present, SUFU accumulates in the cilium but is released from GLI and converted it into the activator form, which permits GLI to enter the nucleus and activate Hh target genes (55, 56). The most important components of the Hh signalling pathway, including PTCH1, SMO, GLI and SUFU, have all been confirmed to be enriched in cilia and their regulation associated with ciliary related signalling (52, 57, 58).

Furthermore, numerous studies have revealed that Notch signalling modulates Shh signalling and functions mechanistically by controlling the ciliary localisation of essential elements of its transduction apparatus. Notch activity regulates subcellular localisation of the PTCH1 receptor, which also controls the translocation of SMO to the primary cilia and its downstream signalling processes (59). Additionally, when NOTCH1 is activated, it causes considerable accumulation of SMO within primary cilia and increased expression of full-length GLI3. Elongation of primary cilia is stimulated by Notch activity *in vitro* and *in vivo* (60). Finally, suprabasal epidermal cells exhibit an enhancement of Notch signalling components at their primary cilia (61).

### 2.2 Primary cilia mediated cellular machinery

Primary cilia play a crucial role in signalling pathways that are essential for many cellular processes. Through the Hedgehog pathway, primary cilia regulate cell proliferation, differentiation and tissue patterning during embryonic development. In the Wnt pathway, primary cilia modulate cellular processes such as cell fate determination and migration by controlling the localisation and activity of key signalling molecules. Additionally, primary cilia play a role in maintaining cellular and tissue architecture by influencing the cytoskeleton and cell polarity. Furthermore, primary cilia are involved in the regulation of autophagy, a cellular degradation process, by coordinating signalling pathways that respond to nutrient availability. The versatile functions of primary cilia underscore their critical role as integrators of signalling networks, orchestrating a wide array of cellular activities essential for organismal health and development. Here, we will introduce key cellular processes regulated by primary cilia, which might contribute to carcinogenesis.

#### 2.2.1 The role of PC in autophagy

The process of autophagy involves recycling intracellular material to preserve tissue and cell homeostasis. It removes non-functional or redundant organelles as well as protein aggregates to generate intracellular nutrients and energy and to eliminate cellular damage (62, 63). Degradation of macromolecules and organelles occurs in the autolysosome, which is formed by the fusion of autophagosomes with the lysosome (64, 65). The main machinery involved in the development of autophagosomes consists of 15 autophagy-related proteins (ATG) (66). The basal body of the primary cilium was found to include ATGs (67).

It is well known that serum starvation of culture cells promotes ciliogenesis as well as autophagy. The first association between these two cellular mechanisms was described in kidney epithelial cells isolated from *Ift88^−/−^* mice and mouse embryonic fibroblasts (MEFs) that had been depleted for *Ift20* (67). As a result, ciliogenesis was impaired, which inhibited autophagy during serum starvation. The requirement of cilia for autophagy was confirmed by findings that inhibition of cilia resorption occurred following starvation-induced autophagy. Wang et al. also provided evidence supporting the beneficial role of cilia in autophagy activation, showing that in human kidney proximal tubular cells, cilia shortening caused by IFT88 knockdown hampered autophagy by triggering mTOR signalling (68).

On the other hand, autophagy can also influence ciliogenesis and cilia length. Autophagy contributed to the degradation of the OFD1 protein’s centriolar satellite pool, which is located near the basal body (69). *Atg5^−/−^* MEFs with normal *Ofd1* levels had a significant reduction of primary cilia, accompanied by considerable shortening but no effect on the cell cycle. Furthermore, ciliogenesis could be restored by *Ofd1* knockdown in both the MEFs and MCF7 breast cell lines, even when serum was present. This suggests that OFD1 suppresses ciliogenesis followed by suppression of autophagy (69). Due to partial IFT20 degradation caused by autophagy, ciliogenesis was negatively regulated in *Atg5^−/−^* MEFs (67). Furthermore, the autophagic degradation of MYH9 mediated by NIMA-related kinase 9 (NEK9), increased actin dynamics, which promoted ciliogenesis. Additionally, it was demonstrated that autophagy mainly stimulates ciliogenesis by depleting OFD1 and NEK9–MYH9 (70). Moreover, resorption of primary cilia and autophagy suppression in serum-starved human retinal pigment epithelial cells was confirmed (71). Similarly, in both a mouse kidney cell line and mouse kidney tissue, autophagy stimulation was able to cause cilia to extend, while autophagy inhibition resulted in cilia shortening (68). Thus, autophagy and primary cilia have a reciprocal relationship in which autophagy is required for ciliogenesis, and PC are needed for autophagy.

Autophagy is considered to be a protective mechanism against cancer development because of its function in sustaining cellular and genomic integrity. On the other hand, autophagy is required for tumour growth once cancer is established because it provides tumour cells a substrate for biosynthesis and energy and defends them from cell death (72, 73). Primary cilia are lost in the majority of cancer cells; nevertheless, some cancer cells, such as pancreatic ductal adenocarcinoma tumour cells, are still able to exhibit significant levels of autophagy (74, 75). Therefore, while the activation of autophagy and the presence of cilia can collectively influence carcinogenesis, their effects on cancer development are specific to the various tumour contexts in which they occur (24).

The effort was performed to identify prognostic autophagy-related genes (ARGs) and AR-lncRNAs to predict clinical outcomes in HNSCC (76) and indeed six differentially expressed ARGs (CXCR4, MAP2K7, RAB5A, ST13, MYC and SAR1A) and 13 AR-lncRNAs were identified. A quantitative proteomics analysis demonstrated that Nuclear protein 1 (NUPR1) is most significantly enhanced in patient samples with OSCC (77). Through a direct increase in transcription factor E3 activity, it was found that NUPR1 maintained autophagic flux and lysosomal functions, hence promoting OSCC cell proliferation and metastasis both *in vitro* and *in vivo*. The NUPR1–TFE3 axis regulates the autophagic machinery during the progression of OSCC.

#### 2.2.2 The role of PC in the epithelial-mesenchymal transition

A process known as epithelial-mesenchymal transition (EMT) allows an epithelial cell to take on a variety of mesenchymal phenotypes. The cytoskeleton, cell-cell junctions and apicobasal polarity of epithelial cells are rearranged during this transdifferentiation process (78, 79). Although EMT is necessary for numerous physiological functions, epithelial cells also undergo it during disease progression in a number of pathological conditions (80). It has been demonstrated that the activation of EMT increases the invasiveness and stemness of cancer cells (81, 82). EMT activation in epithelial cells has been associated with significant alterations in TGFβ, RTK, Wnt, Notch or Hh signalling pathways, which are mostly regulated by the primary cilium (83, 84). According to recent investigations, EMT programs control ciliary signalling and primary ciliogenesis (85–89).

Primary cilia are assembled by Slug-expressing basal cells that are in an intermediate EMT transition state, according to an analysis of ciliogenesis in mouse and human mammary glands (85, 88), and cilia are quite uncommon in Slug-negative cells. Slug (Snail2) is a zinc‐finger transcription factor action that mediates EMT by inhibiting the transcription of E-cadherin, which is responsible for cell adhesion and migratory potential (90). Primary ciliogenesis can be induced in cultured mammary cells by EMT-transcription factors (Snail, Zeb1, Twist) or the absence of E-cadherin (85, 88). In addition, it was demonstrated that EMT-TFs promote the expression of genes encoding positive regulators of ciliogenesis, such as core IFT regulators (88). Another tissue in which EMT is linked to primary cilia during development is the epicardium, where smooth muscle cells forming coronary vessels develop from epicardial cells via the EMT process (91). The suppression of primary ciliogenesis can occur in the mouse epicardium as a result of genetic inactivation of the ciliogenesis inducer WDPCP (86), the protein which is localised in the basal body of PC and recruits IFT proteins to facilitate ciliogenesis (92). Primary cilium ablation impairs EMT and the motility of epicardial cells (86), leading to the failure of proper epicardial cell development into the coronary vasculature, which results in abnormalities of the coronary arteries.

Primary ciliogenesis and EMT are also associated with normal development of the retinal-pigmented epithelium (RPE) of the eye (93). In RPE, ciliogenesis is stimulated during embryonic development and suppressed during tissue maturation in the postnatal stage. The induction of partial EMT in RPE cells has been linked to RPE maturation defects in mice caused by genetic inactivation of the ciliary signalling gene BBS8. At a developmental stage when ciliogenesis is typically inhibited during epithelium maturation, partial EMT is linked to enhanced ciliogenesis in RPE. Contrary, another study demonstrated that lack of primary cilium causes epithelial EMT of kidney tubule cells (94) and TGFβ treatment decreased ciliary length along with EMT induction. *Arl13b* and *Ift20* knockdown resulted in decreased cilia length and increased expression of EMT markers, including collagen III, fibronectin, and a-SMA. Additionally, *Arl13b* and *Ift20*-knockdown displayed higher levels of TGF-β-induced EMT compared to control cells.

EMT and primary ciliogenesis have also been associated in several cancers (85, 87, 89, 95). EMT stimulates ciliary signalling and primary ciliogenesis to encourage claudin-low breast cancer (85). An aggressive form of kidney cancer is represented by a subgroup of renal cell carcinomas (RCCs) that contain a high number of cells that activate EMT programs and primary ciliogenesis (87). In urothelial bladder tumours, EMT is associated with ciliogenesis, especially in invasive cancer cells (95). Recent study has documented that ciliogenesis in glioblastomas is also associated with the activation of EMT transcriptional programs (89).

Global expression profiling studies in independent HNSCC cohorts revealed the presence of a unique patient subgroup with a mesenchymal-like gene expression profile (96). HPV-related and non-HPV-related subgroups with strong immunological and mesenchymal characteristics were described (97). These inflammatory/mesenchymal groups exhibited elevated expression of mesenchymal genes like vimentin and matrix metalloproteinases (MMP9) and downregulation of epithelial markers including P-cadherin and cytokeratins, which both together indicates the process of EMT. However, the association of these changes to disruption of ciliogenesis in this group of tumours have not been evaluated yet.

#### 2.2.3 The role of PC in angiogenesis

On endothelial cells, cilia have been seen both *in vivo* and *in vitro* (98). The primary cilia extend from endothelial cells into the blood vessel lumen, where they detect blood flow and, upon activation, initiate the generation of nitric oxide and calcium signalling (99). Primary cilia on endothelial cells forming the cranial vasculature (78), as well as the caudal artery and vein (100), have been identified in developing zebrafish embryos. On the other hand, endothelial cilia are not necessary for the growth and remodelling of the vasculature in both juvenile and adult zebrafish (101). It has been demonstrated that endothelial cilia are involved in the sensing of vascular remodelling and shear stress during the development of the retina (102). An increased incidence of intracranial haemorrhage was found in zebrafish embryos with mutations in the *Ift* gene that either lack or have disrupted cilia (103–105). In mice, intracranial aneurysms, a sign of disrupted cerebral-vascular integrity, are caused by the loss of components of cilia biogenesis (106). This suggests that endothelial cilia may be involved in preserving vascular integrity throughout development (78). Although endothelial cilia are present in the hyaloid arteries of larval zebrafish, it has been determined that these cilia are not required for the early integrity of the blood-retinal barrier in IFT mutants (107).

Furthermore, deletion of centrosomal protein 41 (CEP41) in zebrafish and human cell lines caused vascular dysfunction, suggesting a pro-angiogenic function for CEP41 (108). The disintegration of cilia, which is involved in the migration and tubulogenesis of endothelial cells, depends on the appropriate regulation of tubulin glutamylation by CEP41. When ciliary tubulin glutamylation occurs in endothelial cells in response to shear stress or hypoxia, CEP41 triggers Aurora kinase A (AURKA), upregulates the production of vascular endothelial growth factors VEGFA and VEGFR2 and causes deciliation. Moreover, CEP41 triggers Hypoxia-inducible factor (HIF1α), which initiates the AURKA-VEGF pathway in hypoxia-induced angiogenesis. The results highlight the significance of ciliary tubulin glutamylation in mechanosense-responsive endothelial cell dynamics and indicate the CEP41-HIF1α-AURKA-VEGF axis as a major molecular mechanism of angiogenesis (108).

Despite the evidence supporting the role of primary cilia in regulating developmental angiogenesis, little is known about how primary cilia may regulate angiogenesis, which includes the growth of new blood vessels into solid tumours. Further study evaluating their association during tumorigenesis will be necessary, and AURKA-VEGF signalling could represent a promising future target.

#### 2.2.4 The role of PC during hypoxia

Hypoxia, a typical microenvironmental feature of solid tumours, is characterised by fast-growing, highly proliferative cells away from blood arteries, which prevents oxygen from diffusing in such a weakly vascularised tissue (109). Tumour cells can execute an adaptive response under these unfavourable conditions, which leads to the reprogramming of cellular metabolism, stimulation of cell proliferation, resistance to apoptosis, limitless potential for replication, activation of angiogenesis, avoidance of immune attack and migration towards less hypoxic environments and invasion. Hypoxia-inducible factors (HIFs) are the primary controllers of oxygen homeostasis and play a role in coordinating all these responses to the hypoxic environment (109, 110). In a previous investigation on mesenchymal stem cells (MSCs), it was shown that elongated primary cilia in hypoxic environments were lost over time (111). HIF-1α gene silencing prevented hypoxic cultures from losing their cilia, while primary cilium growth was reported to be reduced in MSCs, which express constitutively active HIF-1α. Similarly, when tendon cells maintained in normoxic versus hypoxic conditions were compared, a significant decrease in the percentage of extended cilia was observed in the hypoxic group (112). By contrast, HIF-2α was found to accumulate in the ciliary axoneme in mouse neural cells and stimulate ciliary elongation in hypoxic conditions (113), and HIF-2α interacted with IFT88 to affect ciliary signalling during hypoxia. These contentious outcomes could be partly attributed to distinct cell types and the constant antagonistic roles of HIF-1α and HIF-2α (114).

Moreover, the primary cilium is maintained via an interconnected signalling cascade that includes GSK3β and pVHL (von Hippel-Lindau) in renal cysts (115). The best understood role of pVHL is its involvement in the oxygen-dependent ubiquitin-mediated proteasomal degradation of HIFα subunits (116). In renal cell carcinoma (RCC), the expression of VHL was directly associated with the production of cilia (117, 118), while VHL was necessary for ciliogenesis regardless of the HIFα level (117). On the other hand, HIFα plays a major role in mediating the effects of VHL on the primary cilium in the case of renal cysts (119). It is still unknown how HIFs mediate cilia resorption. One possibility is that pVHL controls the activity of Never in mitosis gene A (NIMA)-related kinase 8 (NEK8), which is critical for cilia and the cell cycle (120). NEK8 was overexpressed in RCC cell lines lacking pVHL. The results indicated that NEK8 expression was elevated in the hypoxic environment, suggesting a potential involvement of HIFs in its control. Furthermore, primary cilia resorption resulting from pVHL knockdown was impeded by downregulation of NEK8. Moreover, the inactivation of VHL induces AURKA due to the stabilisation of HIF1 and 2 and subsequent regression of primary cilia (121). To conclude, HIFs can mediate the disassembly of primary cilia via different pathways and their involvement in ciliogenesis may play a crucial part in tumour cells’ ability to adapt to the hypoxic microenvironment, ultimately resulting in tumour growth.

Moreover, recent studies on hypoxia gene sets have demonstrated their efficacy as prognostic and predictive markers in HNSCC patients (122), and a unique hypoxia score that uses methylation patterns to anticipate HNSCC prognosis was defined (123). However, the classifier’s effect was restricted to HPV-negative tumours, which will be necessary to follow further.

#### 2.2.5 The role of PC in stemness

The mechanisms of stemness maintenance are still not fully understood. However, emerging evidence suggests that these processes involve the function of the primary cilium. Many adult stem cells (ASCs) and/or progenitor cells have been described as forming primary cilia, raising a great deal of attention to their functional roles in these cells (124). Under carefully controlled circumstances, ASCs can differentiate *in vitro* into distinct cell types. Mesenchymal stem cells (MSCs) cannot properly differentiate into osteoblasts and adipocytes *in vitro* when their primary cilia are disrupted (125). Moreover, it has been demonstrated that ciliary Hh signalling mediates the neuronal-like differentiation of MSCs, indicating the cilium’s role in this process (126). Remarkably, altered ciliary structure was demonstrated during cell differentiation (44, 127–129). It has also that MSC pluripotency and proliferation depend on primary cilia-dependent signalling. Following deciliation, the expression of the stem cell markers *Oct4, Nanog* and *Sox2* was significantly decreased (130). Parallel to this, when AURKA was inhibited, adipose-derived MSCs exhibited higher expression of *Oct4, Nanog* and *Sox2* (131, 132), and restoring primary cilia function highlighted the pivotal function of the primary cilium in controlling stemness in both diseased and physiological conditions (133).

Well-coordinated cell signalling is necessary for the correct temporal and spatial activation of stem cells. It has been demonstrated that ciliary dynamics in stem cells depend on the appropriate functions of Prominin-1 (Prom1/CD133), a cholesterol-binding membrane glycoprotein (134). The lack of *Prom1* disrupts stem cell quiescence maintenance and activation by reducing ciliary dynamics and eliminating the growth-stimulating effects of SHH treatment. Thus, PROM1 appears to be a crucial regulator sustaining the proper responsiveness of stem cells to external stimuli.

While cilia are necessary for normal stem cells to remain in the quiescent state, they are frequently lost in proliferative progenitors. Determining the ciliation state of cancer stem cells is therefore crucial. In human biopsies (135) and mouse models (136, 137), the loss of PC in basal and luminal cells/progenitors in breast cancer has been extensively described. According to a study on medulloblastoma, non-ciliated cells, even those with constitutively active SMO, could not develop tumours, whereas ciliated precursor cells lacking PTCH1 were capable of developing tumours (138). Nevertheless, cilia demonstrated tumour-suppressive activity when the Hh pathway was dysregulated downstream of SMO/PTCH1. Likewise, it is possible to categorise glioblastomas (GSCs) and rhabdomyosarcoma (RMS) as independent and cilia/Hh-dependent cases (139, 140). When RMS arises from ciliated, undifferentiated myoblasts, the cancer cells will be dependent on Hh; on the other hand, if RMS originates from more differentiated progenitors, it will be Hh independent due to the absence of cilia. Primary cilia ablation generally increases proliferation while significantly suppressing Hh signalling, whereas cilia restoration stimulates GSC differentiation and reduces proliferation (141, 142). To conclude, the stage at which the Shh signalling cascade is dysregulated seems to determine whether PCs are present in precursor cells and how this influences tumour growth.

A recent study by Luo et al. analysed the role of cancer stemness in HNSCC (143), where tumour samples had more stem-like characteristics than non-tumour samples. In HNSCC, the stemness score was not a reliable indicator of prognostic prediction, although it was crucial and associated with tumour aggressiveness. When the high-SPI (stemness-related prognostic index) HNSCC group was compared to the low-SPI group, several significantly up-regulated pathways associated with tumour formation and progression were uncovered, such as angiogenesis, hypoxia and EMT signalling. The high-SPI group and the low-SPI group differed significantly, indicating the usefulness of SPI in detecting patients with HNSCC (143).

## 3 Alteration of primary cilia in cancer

The hallmarks of cancer cells and neoplastic development include deregulation of the cell cycle and uncontrolled cell proliferation. The cell cycle appears to be the principal regulator of primary cilia formation, with the centrosome serving as a crucial checkpoint. In a quiescent state in cells, the centrosome functions as a template for cilia assembly. When the cell is dividing, the cilium is reabsorbed, and the centrosome serves as a microtubule-organising centre (18, 24). Numerous cell cycle regulators, including Polo-like kinase 1 (PLK1), Aurora kinase A and Never in mitosis A (NimA)-related kinase 2 (NEK2), have been found to play a critical role in cilium fate; moreover, they are associated with tumorigenesis. This highlights the interplay between ciliogenesis and cell division (24). Additionally, IFT88 has been shown to be essential for the formation of primary cilia and to be a major regulator of the G1-S transition in non-ciliated cells (144, 145). As a result, cilia dysfunction may cause uncontrolled entry and progression of the cell cycle in tumour cells (146). On the other hand, persistence of the primary cilium can stop the cell cycle and force the cells to turn into quiescence (24). Therefore, primary cilia have the potential to be targeted in tumours to promote their regeneration and restore the physiological rate of proliferation (300).

Primary cilia were found to be lost or disassembled in several tumorous tissues, including renal (147), pancreatic (74), colorectal (146), cholangiocarcinoma (148), breast (135), ovarian (149), prostate (150), astrocytoma/glioblastoma (151), melanoma (152), chondrosarcoma (153) and oesophageal SCC (154, 300). Whether this lack of cilia is the cause or the consequence of tumour transformation is not well understood. On the other hand, some cell subpopulations within human glioblastoma tumours were found to be ciliated (155), and both plexiform and follicular ameloblastomas have an abundance of primary cilia (156). Similarly, the frequency of primary cilia was noticeably higher in adenocarcinoma of the lung, colon and pancreas and follicular lymphoma (157). Ciliated adenocarcinomas and SCCs have been reported in the oesophagus and tonsils (158, 159). Twenty-five percent of cancer cells in patients with pancreatic ductal adenocarcinoma were found to have primary cilia. The prognosis was worse for patients with primary cilia positivity because they had a higher rate of lymph node metastasis (160). The presence of primary cilia has also been revealed in the spindle and epitheloid gastrointestinal stromal tumour cells (161, 162), in basal cell carcinoma (57) and in several human cancer cell lines (163). Interestingly, in medulloblastoma or basal cell carcinoma, primary cilia are present or absent depending on the signalling pathway, which is currently activated (57, 138). Therefore, evaluation of their occurrence alone is not sufficient to predict tumorous tissue behaviour. Moreover, with the improvement of methodological possibilities of cilia detection and investigation of their role, there is an increase in new evidence of primary cilia’s existence in tumours, which were previously characterised as missing cilia (163). Also, the length of the cilia should be assessed in cancer cells in comparison to normal tissue as abnormal ciliary size can indicate affected signalling (164). Furthermore, certain cancer tissues were shown to have structural anomalies of primary cilia, such as extension of the axoneme, numerous basal bodies and axoneme branching (157).

Focusing on HNSCC, a significant decrease in ciliated cells number was found in oral leucoplakia, and almost no ciliated cells were reported in OSCC when these tissues were compared with normal oral epithelium or tissues adjacent to the tumour (165). Nevertheless, more detailed analyses focused on possible differences in the frequency of primary cilia in individual regions of the oral cavity, and their association with tumour grading are still missing; therefore, it will be necessary to follow this topic in detail in the future.

Primary cilia do not occur on the cell at the time of their division. As many cancers exhibit a very high mitotic rate, the lack of primary cilia in these tumours could lead to the misleading assumption of disruption of cilia function, but their absence can be simply a consequence of observed high cellular turnover. On the other hand, renal and pancreatic cancer cells do not contain primary cilia independently of any decreases or increases in Ki67 staining (a cell proliferation marker) (74, 118). Moreover, in histological subtypes of ameloblastoma, an increased number of cilia with completely different mitotic rates was found (156). This would support the idea that the loss of cilia in certain cancer types is not associated only with the cell proliferation rate, indicating the necessity of evaluating the number and morphology of primary cilia together with their function and associated signalling to be able to uncover the cellular processes that contribute to tumour initiation and progression. In addition, an increase in the quantity and/or length of primary cilia or cilia fragmentation led to resistance to targeted therapies in several experimental models (166). Consequently, it will be important to determine whether particular cilia frequency levels are correlated with specific cancer subtypes as well as with clinical information such as survival, recurrence and treatment response (150).

Alteration of cancer cell ciliation as well as changes in the tumour microenvironment (TME) during tumour growth enhance asymmetric intercellular signalling (167). The TME, which is made up of immune and stromal cells that enter the tumour through lymphatic and blood vessels, comes into contact with tumour cells throughout this process (168). As tumorigenesis progresses, its surrounding microenvironment and tumour cells interact, undergo adjustments continuously and evolve characteristics that support development, invasion and metastasis (169, 170). While endothelial cells and fibroblasts within the TME probably have more cilia than cancer cells, lymphocytes and myeloid cells that infiltrate the TME typically do not. This uneven cilia distribution between the cancer cells and surrounding non-cancerous cells affects paracellular signalling in the TME because primary cilia play a crucial role in transducing different signalling pathways (168). Hypoxia is a well-known feature of the TME that is directly related to angiogenesis, cell migration, proliferation and the tumour immune response, all of which promote tumour growth and have a negative impact on prognosis (171). The number of cellular signals associated with the cancer environment, such as inflammatory cytokine and metabolic signals, have been associated with the primary cilia (172). Proinflammatory cytokine IL-1 is able to induce cilia elongation (173). Conversely, proinflammatory cytokine IL-6 diminishes the number of primary cilia (174), similar to tumour necrosis factor-alpha, which can cause loss of primary cilia in a dose-dependent manner (172). These results indicate that ciliary assembly is regulated as a response to inflammatory cytokines. Additionally, metabolic factors in the environment can affect cilia growth as primary cilia formation was found to be regulated via glucose deprivation (175), and primary cilia in hypothalamic neurons have been revealed to be essential for the sensing of metabolic signalling (176). Moreover, tumour‐associated lipogenesis in prostate cancer cells may interfere with cilia formation, impede environmental sensing, cause abnormal cell signalling and disrupt polarised tissue architecture (177). In summary, an understanding of primary cilia function not only in cancer cells but also in cells within TME and their role in signalling could identify novel cancer pathogenic factors related to primary cilia disruption and potential anticancer therapies.

Moreover, it is necessary to consider the interactions between the surrounding tissue matrix and immune cells that comprise the TME, which are probably the cause of the high rate of metastatic HNSCC and recurrence (178). Interestingly, HNSCCs are immune-suppressive in addition to being able to evade immune cell detection (179, 180), and immunosuppression can be induced in the TME of HNSCC, which ultimately leads to the disease’s progression (181, 182). These factors induce the dysfunction of numerous immune killer cells and aberrant release of immune-related substances. An essential process that allows cancer cells to escape immune cell attack in patients with HNSCC is the formation of immune checkpoints, which are highly expressed in the TME (183, 184). However, immune-checkpoint-blocking treatments for HNSCCs have demonstrated a limited favourable safety profile and response rate in recent years (185–188). Therefore, further studies are needed to optimise HNSCC treatment in order to effectively incorporate the response of all interacting TME components to improve the prognosis of patients.

Additionally, anticancer drugs are dispersed through tissues during cancer treatment. As small molecule medications can easily penetrate the circulation and frequently alter the structure and function of cilia as well as signalling, it is important to consider how these drugs may affect immune system activity as well as the cilia of endothelial cells in the blood and lymphatic vessels. Because of this, the curative potential of cancer treatment may be weakened or even counterproductive due to side effects (168). Recently, it was reported that primary cilia influence endothelial permeability by controlling junction protein expression and localisation (189), which can be connected with the infiltration of circulating tumour cells that cause metastasis. So, it is important to address whether the effectiveness of these anticancer compounds is increased or decreased by counteracting ciliary function in certain tumours (168). To ensure that medications are used precisely and accurately in these cancer patients, additional investigation and validation of cilia dynamics and related signalling may be necessary.

## 4 Cilia-related signalling pathways in squamous cell carcinoma of the head and neck region

Considering the primary role of the cilium in the fine-tuning of signalling pathways that are often dysregulated in cancer (19) as well as its close connection with the cell cycle (190), these organelles might be decisive in the biology of cancer (19). Malfunctioning cilia may result in abnormalities in signalling, leading to the induction of tumorigenesis. Depending on the type of cancer, the cancer-initiating mutations and the altered molecular pathways, the presence or absence of the primary cilium can either accelerate or slow down the progression of the disease (24). Among the signalling pathways associated with the primary cilium, the PDGF, Wnt and Hedgehog pathways are well-studied (24).

Here, we first introduce information regarding alterations in signalling pathways in different tumour types including presenting the role of these cilia-associated signalling pathways in the pathogenesis of squamous cell carcinoma in the head and neck region.

### 4.1 Platelet-derived growth factor signalling in HNSCC

PDGFs and their receptors are expressed in a variety of malignant tumour cells and organs, including pancreatic cancer (191), hepatocellular carcinoma (192), breast cancer (193) and ovarian carcinoma (194). Their expression levels are associated with invasiveness, tumour growth, chemoresistance and poor clinical outcomes (195, 196). Disrupted PDGFRα signalling in ovarian cancer cells was correlated with a loss of primary cilia (149). Increased PDGF expression was also observed in OSCC (**Table 1**), where it contributes to tumour progression and metastasis (212, 216–218). Augmented PDGF signalling was predicted to be the risk factor for OSCC (212) and in OSCC patients, there is a favourable correlation between increased PDGFRα overexpression and metastasis as well as decreased survival (210). Moreover, the interaction of the PDGF-BB ligand with the PDGFβ receptor induces the formation of cancer-associated fibroblasts in OSCC (219).

**Table 1:** Expression of selected cilia-associated genes in OSCC.

Gene	mRNA	Reference	Protein	Reference
SHH	Up	(197, 198)	Up	(15, 199–203)
PTCH1	Up	(204)	Up	(198, 199, 201, 203, 205)
SMO	Up	(198, 204)	Up	(198, 199, 201, 206)
GLI1	Up	(204)	Up	(199, 200, 205, 206)
GLI2			Up	(199, 201)
EGFR	Up	(165)	Up	(165, 207–209)
PDGFRA, PDGFRB	Up	(210)		
PDGFA	Up	(211, 212)	Up	(211)
PDGFB	Up	(211, 212)	Up	(211)
CTNNB1	Up	(213)	Up	(213)
FZD2	Up	(214)		
GSK3B	Up	(215)		

### 4.2 Epidermal growth factor signalling in HNSCC

Under normal physiological conditions, epidermal growth factor (EGF) regulates several cellular processes, including cell growth, migration and differentiation. Due to *EGFR* gene amplification, protein overexpression, mutations or in-frame deletions, EGFR signalling is frequently altered in a number of human malignancies, including glioblastoma, brain, lung, breast and ovarian cancers (220, 221). EGFR overexpression is a risk factor for HNSCC that is present in around 90% of patients (222). Particularly, EGFR was described to be overexpressed in OSCC (**Table 1**) (165, 207). Moreover, a positive correlation has been found between its overexpression and an earlier relapse and a lower overall survival. Studies on OSCC patients revealed amplifications of the EGFR gene (13, 209, 223) that were associated with perineural invasion and extracapsular spread (13). In oral carcinoma, an *EGFR* mutation on exon 19, codon 746 (Glu746del) was found (224).

### 4.3 Wnt signalling in HNSCC

Wnt signalling disruptions have been reported in a number of cancer types, including colorectal, lung, prostate and breast cancer (225). In colon cancer cells, the loss of the primary cilium leads to hyperactivation of Wnt signalling (24). It has been demonstrated that cilia loss amplifies pro-tumorigenic WNT/β-catenin signalling, which is enough to cause benign cells to develop metastatic melanoma (226). Human prostate cancer has also been shown to lack primary cilia. High levels of nuclear β-catenin expression, indicating Wnt pathway activation, were observed in unciliated cells (150). By contrast, in the WNT group of medulloblastoma, primary cilia promote tumorigenesis by supporting the synthesis of β-catenin (227). Wnt/β-catenin signalling was also found to be upregulated in oral tumours (213, 228, 229). Abnormal activation of Wnt/β-catenin signalling was proposed to induce tumour formation and metastasis in HNSCC (230) as well as in OSCC (213). However, crosstalk with other molecules that can result in pathway cross-activation appears to be the reason for the higher rate of Wnt/β-catenin pathway activation in HNSCC. It has been demonstrated that increased EGFR signalling, one of the most commonly dysregulated signalling pathways in HNSCC, can activate β-catenin. In this context, delocalised β-catenin expression was linked to increased EGFR expression (231). In a different study, there was found a correlation between high expression of EGFR in OSCC samples and the nuclear translocation of β-catenin (232).

### 4.4 Hedgehog signalling in HNSCC

A number of cancer types have been linked to aberrant Hh pathway activation, including lung, breast, prostate and basal cell carcinoma (233). In human tumours, the Hh pathway is disrupted either through mutations of signalling proteins such as SMO, PTCH1 and SUFU or through overexpression of HH (51). In oesophageal squamous cell carcinomas, two somatic mutations in PTCH were detected. Among these alterations a missense mutation (CAG to CTG, Gln to Leu at codon 816) in exon 14 and a nonsense mutation (CAG to TAG at codon 361) in exon 8 were uncovered (234).

Studies in mouse models have determined that cilia play a role in both promoting and inhibiting cancer through their mediation of Hh signalling (57, 138). The Hh pathway is “on” when Hh is present because GLI proteins are processed into the activator form, whereas in the absence of ligand, this pathway is “off” when GLI proteins are processed into the repressor form. Furthermore, inhibitors of SMO and GLI regulate Hh signalling in the presence of cilia, whilst overexpression of Hh signalling may occur if cilia are lacking or malfunctioning (57). Ablation of primary cilia by *Kif3a* or *Ift88* deletion prevented the growth of medulloblastoma tumours in animal models which was induced by a constitutively active SMO. On the other hand, in medulloblastoma models with constitutively active GLI2, primary cilia ablation accelerated tumour growth (138). Similarly, ciliary ablation in basal cell carcinoma (BCC)‐like tumours, induced by an activated form of SMO, inhibited the growth of carcinoma, conversely, in tumours induced by activated GLI2, carcinogenesis was accelerated (57). In other words, mice with wild-type cilia and active SMO produced medulloblastomas and BCCs, whereas mice with mutant cilia and active SMO did not develop tumours. Tumours could not develop because the active SMO needed cilia to initiate the Hh pathway. Tumorigenesis was markedly increased in the absence of cilia when the Hh pathway was stimulated downstream of cilia by ectopically producing an activator form of GLI. On the other hand, cilia facilitate the production of the GLI protein’s repressor form, which is expected to counteract the activator form of GLI protein that is expressed exogenously and inhibit the growth of tumours (57, 138). In the case of an ovarian tumour, the reduced frequency of primary cilia in cancer cells may cause abnormal Hh signalling and promote ovarian carcinogenesis (149). By contrast, the elimination of primary cilia is sufficient to suppress Hh signalling and the tumour-forming potential of mammary tumour-initiating cells (85). Therefore, the ability of primary cilia to modulate carcinogenesis may depend on the Hh signalling pathway’s conditions. Given that cilia can play a dual role during tumorigenesis, it is crucial to determine which step of the Hh pathway is disrupted and analyse the status of primary cilia in tumours.

Recently, it was shown that Hh signalling promotes cancer cell proliferation, malignancy and metastasis (49) as well as several neoplastic transformations (235–237). In HNSCC, the molecules of the Hh signalling pathway are significantly overexpressed when compared to those of the normal oral mucosa (202, 238). It is possible that this signalling is implicated in the development of tumours because of a significant increase in the expression of Hh pathway members and their progression that is seen from the normal oral mucosa through dysplasia up to HNSCC tissue (201). Moreover, low expression of GLI1 was detected in the non-neoplastic oral mucosa near the tumour, and the clinical stage of the patients was also closely linked with the expression level of SMO (204). High SHH and PTCH1 expression was found in the parenchyma of the endophytic type of OSCC. PTCH1 expression was also located in macrophages and alpha-smooth muscle actin-positive fibroblasts in cancerous stroma. Therefore, increased Shh signalling influences not only epithelial compartments to growth but also affects stroma cells (203). Advanced HNSCC tumours were shown to express higher levels of SHH, PTCH1, SMO, GLI1, GLI2 and GLI3 (**Table 1**). This suggests that the Hh pathway plays a critical role in the growth and invasiveness of these malignancies (15, 200, 205, 206, 239). Overexpression of SHH and members of this pathway was also described in several cell lines of human OSCC, and Shh signalling was found to be involved in increased cell proliferation of tumorous cells (240). In agreement with these findings, SHH produced by stromal fibroblasts of SCC supported the proliferation and migration of cancer cells (241).

Furthermore, a correlation between Hh expression and lymph node invasion suggests that Hh signalling plays a role in the development and spread of HNSCC (15, 200, 205, 206, 239). In addition, the correlation between Hh pathway components and epithelial-mesenchymal transition also promotes the significance of the Hh pathway during metastasis of HNSCC (200), and enhanced Shh signalling can also induce neoangiogenesis (203). Furthermore, a greater histological grade of the tumour was associated with overexpression of GLI1, GLI2, PTCH1 and SMO, which may indicate a critical function for Hh signalling in HNSCC malignancy (198, 204, 206, 242). Interestingly, oral and oropharyngeal SCC tumours with a higher Broders’ grade (poorly differentiated tumours) were GLI1 negative and exhibited high expression of PTCH1, while differentiated tumours exhibited high GLI1 (198). Higher expression of SHH, PTCH1, SMO and GLI1 was considerably linked with lower overall survival, as reported by several studies (15, 199, 200, 205, 243); similarly, an increased level of SUFU indicated a worse prognosis in oral cancer patients (244). As inhibition of the Hh signalling pathway can suppress the self-renewal of cancer stem cells and their drug resistance in OSCC (242), it has the potential to inhibit the induction of cancer invasion in these cases (203).

In summary, Hh signalling is activated in HNSCC and is assumed to mediate tumorigenesis. Moreover, the presence or lack of cilia may significantly affect the efficacy of targeting distinct Hh signalling members, depending on which component of the pathway functions to maintain carcinogenesis (51). However, verification of how exactly cilia are linked with members of relevant signalling pathways is necessary to determine and if there is a direct correlation between cilia and a given human cancer.

## 5 Inhibitors of cilia-related signalling and their potential usage for the treatment of head and neck squamous cell carcinoma

As primary cilia-associated signalling plays a role in the pathogenesis of squamous cell carcinoma in the head and neck region, we paid attention here to the possibility of synthetic inhibitor use to target signalling pathways dysregulated in HNSCC. Moreover, several drugs used for cancer treatment have been shown to exhibit activity regulating cell ciliation (245), which we are also following further.

### 5.1 Targeting of PDGFR signalling

**Figure 2:**
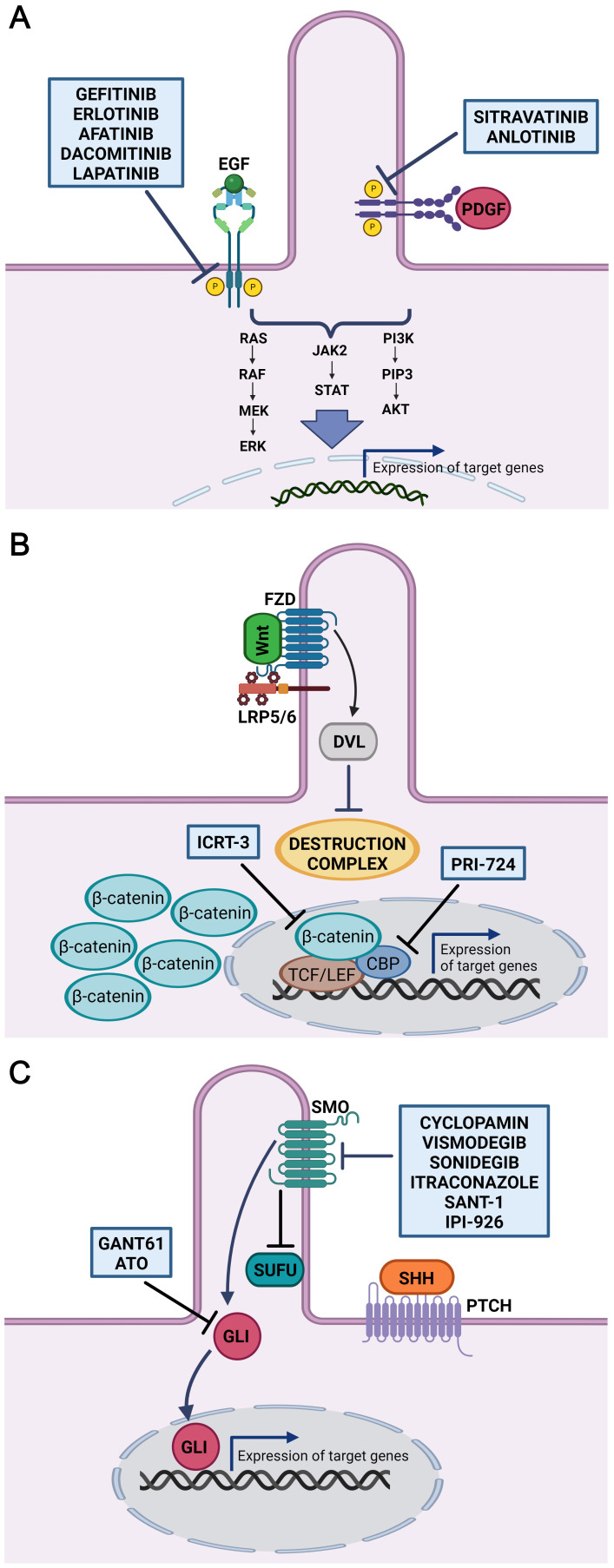
Inhibitors of cilia-related signaling pathways. Schematic overview of inhibitors potentially useful in the treatment of HNSCC (OSCC). **(A)** Inhibitors gefitinib, erlotinib, afatinib, dacomitinib and lapatinib target tyrosine kinase of EGFR. Anlotinib and sitravatinib inhibit tyrosine kinase activity of PDGFR. Thus, wide range of downstream signalling pathways including Ras-Raf-MEK-ERK, PI3K/AKT, and JAK2/STAT involved in regulating many cellular processes is suppressed. **(B)** Inhibitor of Wnt signalling, ICRT-3, selectively inhibit the interaction between β-catenin and TCF/LEF transcription factors. PRI-724 specifically target the interaction between β-catenin and its transcriptional coactivator CREB-binding protein (CBP) thereby inhibiting transcription of Wnt target genes. **(C)** GANT61 and ATO are antagonist of GLI proteins, transcriptional effectors of the Hh signalling pathway. Cyclopamin, vismodegib, sonidegib, itraconazole, SANT-1 and IPI-926 bind directly to Smoothened and inhibits downstream Hh signalling. Figures were created with BioRender.com.

It has been established that an inhibitor targeting PDGFR, sitravatinib (**Figure 2A**) alters the immune and tumour microenvironment in patients with oral cavity carcinomas (246). Another tyrosine kinase inhibitor, anlotinib (**Figure 2A**), exhibited prominent antitumor activity *in vitro* in a human OSCC model. Treatment with anlotinib significantly decreased cell migration, viability and proliferation. On the other hand, anlotinib suppressed PI3K/Akt/Bad phosphorylation and increased apoptosis by upregulating the production of RAS protein (247). Additionally, anlotinib inhibited cell proliferation in OSCC and caused cell cycle arrest, which was accompanied by abnormal spindle apparatus formation. Due to its connection with the cell cycle, the disruption of primary cilia function in this case is highly probable and indicates this signalling pathway as a potential therapeutic target (248).

### 5.2 Targeting of EGFR signalling

A promising target in HNSCC (OSCC) therapy also seems to be the inhibition of EGFR by drug molecules bound either to the extracellular domain or to the cytoplasmic region of the receptor. From these, EGFR monoclonal antibodies displayed favourable but limited effects in HNSCC patients (249), where only 10%–30% of patients responded to monotherapy based on antibody EGFR inhibitors (250). Several early- and late-phase clinical trials are testing drugs in the tyrosine kinase inhibitor class. From these, gefitinib, erlotinib, afatinib and dacomitinib (**Figure 2A**) have demonstrated potential for the treatment of HNSCC (251–254). A study in OSCC cell lines derived from patients with amplified EGFR revealed responsiveness to afatinib, erlotinib, gefitinib, lapatinib and saracatinib (209). When EGFR inhibitors were combined with chemotherapy or radiation therapy, the response was better than if the inhibitors were used alone. This suggests that the functional heterogeneity of cancer stem cells in advanced HNSCC may be connected to tumour resistance to EGFR inhibitor monotherapy (255, 256). Remarkably, in patient-derived HNSCC xenografts, treatment of EGFR inhibitor-resistant HNSCC cells with the Shh inhibitor IPI-926 suppressed tumour growth and prevented tumour recurrence (257). Additionally, it was found that dual targeting of the EGFR and Shh pathways decreased HNSCC cells’ capacity to proliferate and form colonies (258). Moreover, combination therapy that blocked both Shh and EGFR enhanced the clinical results for patients with HNSCC (259).

Another therapeutic approach offers targeting of primary cilia through their interference with EGFR signalling. EGFR kinase can suppress ciliogenesis by stabilising cilia disassembly by kinase Aurora A (260). EGFR activation initiated the absorption of cilia while downregulation of Aurora A rescued cilia formation and reduced the growth of OSCC lines *in vitro*. Based on these findings, the restoration of primary cilia function was proposed as a possible therapeutic target for OSCC patients (165).

### 5.3 Targeting of Wnt signalling

Manipulation of Wnt signalling limits the survival of cancer cells, which are usually resistant to chemotherapy, thus enabling their tumorigenesis to be targeted. Downregulation of β-catenin causes the induction of apoptosis and impedes the cell proliferation and self-renewal properties of OSCC cells (213). Moreover, the inhibitor of β-catenin-responsive transcription (ICRT-3, **Figure 2B**) arrested the cell cycle and reduced the motility of HNSCC cells (261). β-catenin levels in OSCC were decreased by microtubule-targeting drugs (MTAs) that inhibited microtubule dynamics. MTAs offer important antitumor effects due to the decrease in Wnt/β-catenin signalling where combining MTAs with Wnt/β-catenin signalling antagonists may be an effective approach to cancer treatment (262).

Additionally, the effects of combinatorial use of Wnt/β-catenin and Hh pathway inhibitors were tested *in vitro* in HNSCC cells (263). In the present study, combinations of vismodegib (an inhibitor of the Hh pathway; see below) and PRI-724 (a Wnt/β-catenin inhibitor; see **Figure 2B**) significantly reduced cell migration, downregulated transcript level of SCC markers and decreased cell proliferation. Furthermore, PRI-724 and the EGFR inhibitor erlotinib together reduced cell growth and triggered apoptosis (263) indicating high therapeuitic potential.

### 5.4 Targeting of Hh signalling

Because a relatively broad range of tumours is linked to dysregulation of the Hh pathway, targeting of Hh-related molecules seem to be potentially promising anticancer strategies (264–266). The primary focus of therapeutic research for the Hh signalling pathway has concentrated on SMO and GLI1 targeting. Both natural and synthetic antagonists for SMO and GLI1 have been produced (**Figure 2C**), and several of them have demonstrated varied degrees of success in clinical studies. Shh pathway inhibitors mainly target SMO since blocking of SMO stops the downstream activation of GLI. Nevertheless, distinct molecular pathways can also activate GLI transcription factors independently of SHH ligand and SMO, which needs to be considered when assessing the effectiveness of inhibitor treatment (267). Here, we review several Hh antagonists that were recently used in clinical trials and exhibit the potential to be effective in treating oral and neck SCC.

Cyclopamine is a steroidal alkaloid first known for its teratogenicity, causing cyclopia in cattle grazing on pastures rich in lily plants (Veratrum species) (268). By binding directly to the SMO receptor and blocking it, cyclopamine inhibits the Hh signalling pathway. It decreases the growth of cells with aberrant PTCH1 function (269). In OSCC, cyclopamine efficiently reduced GLI expression, slowed down the growth of cells, stimulated G1 arrest, increased apoptosis and prevented OSCC cell migration (270). Its weak solubility, limited potency, quick clearance, non-specific toxicity and chemical instability, however, represent limitations (271). This naturally occurring alkaloid is nowadays replaced by the second-generation synthetic cyclopamine derivative, vismodegib.

Vismodegib (GDC-0449) is a small molecule inhibitor of the Hh pathway. It blocks Hh signalling by binding to the receptor SMO (272). Vismodegib is approved for the medication of adult patients with advanced or metastatic basal cell carcinoma, which cannot be treated by surgery or radiotherapy. A recent study analysed its impact on the radiation sensitisation of cell lines of oral and neck SCC by studying viability, the cell cycle, DNA damage repair, cell death and clonogenic survival in three-dimensional cultures of an HNSCC cell line (273). Vismodegib was shown to decrease the expression of downstream Shh signalling, especially targeting GLI1 and SMO in a cell line- and irradiation-dependent manner in SCC cells. Vismodegib also decreased proliferation in analysed cell lines and reduced cell viability (273, 274). Moreover, inhibition of the Hh pathway by vismodegib decreased osteolytic activity induced by HNSCC (275).

Sonidegib (LDE 225, Odonzo) is a synthetic antagonist of the Hh pathway, which binds to the SMO receptor, and it is primarily metabolised by CYP3A in the liver (276). Sonidegib is in a clinical trial for HNSCC and other advanced stages of solid tumours (clinical trial No. NCT04007744), and it is expected to inhibit tumour growth by blocking key enzymes of cell growth. As the majority of the information about the action of sonidegib is known from studies on basal cell carcinoma (277–279), a detailed evaluation of its possible effect and mechanism of action on HNSCC will be necessary.

Itraconazole is a broad-spectrum antifungal molecule inhibiting lanosterol 14-a-demethylase, an enzyme-producing cholesterol in mammals (280, 281). Itraconazole was shown to regulate the Hh pathway and to prevent its downstream signalling during tumorigenesis, including OSCC (274, 282). It prevents SMO from accumulating in the primary cilia, which is physiologically stimulated by Hh signalling (282). In the CAL27 cell line (OSCC cell line), itraconazole reduced the expression of Hh target genes, diminished cell viability and induced apoptosis (274). Another *in vitro* study revealed that itraconazole caused cell cycle arrest and apoptosis, suppressed cell invasion and migration, and reduced OSCCs’ ability to proliferate and form colonies. Itraconazole suppressed Ki-67 expression, promoted apoptosis, and inhibited tumour growth in the xenograft model generated from OSCC patients. Moreover, itraconazole decreased the Hh pathway’s protein expression in OSCCs (283).

GANT61 is an artificial molecule that originates from hexahydropyrimidine. It was proven to efficiently bind to GLI transcription factors (284). GANT61 diminishes the expression of SHH, GLI1 and PTCH1 in various cancer cell types, including OSCC (270, 284–287). A decrease in the OSCC population, reduced cell viability, promotion of G1 arrest, induction of apoptosis and inhibited migration of cells was observed after its application on cell cultures (270, 288). GANT61 entered a preclinical phase for breast cancer treatment (289) and seems to be a promising molecule for future analyses of its effect on OSCC.

Arsenic trioxide (ATO), a well-studied GLI antagonist, exhibited increased growth-inhibitory and cytotoxic activity in HNSCC cells (290). Reduced GLI1 expression, changes in cell shape and nuclear fragmentation and an increase in apoptosis were the outcomes of ATO treatment for OSCC cells (291). Additionally, ATO decreased the stability of GLI2 transcription factor, inhibiting its accumulation in the primary cilium in response to Shh signalling (292). Furthermore, ATO may make HNSCC more radiosensitive (293), while the combination of chemotherapy with ATO enhances apoptosis in OSCC (294). However, substantial adverse effects are caused by high dosages of ATO, which are necessary to effectively suppress solid tumours *in vivo* (295). Current research focuses on combination therapy using low dosages of ATO and other cytotoxic medicines in light of these findings.

Vorinostat (suberanilohydroxamic acid) is an inhibitor of histone deacetylases (HDAC1, 2, 3 and 6) and exhibits a wide-range spectrum of epigenetic activities (296). HDAC inhibition resulted in acetylation of α-tubulin followed by alterations in cell motility, proliferation, the cell cycle and repair of DNA damage (297, 298). Vorinostat treatment caused modest upregulation of SHH expression in acute myeloid leukaemia cells (AML). Explicit targeting of the Shh pathway by SANT-1 as a high-affinity antagonist of SMO, followed by vorinostat treatment in an AML cell line, induced epi-sensitisation of cells leading to cell death (296). Vorinostat treatment of patients with recurrent HNSCC, which are metastatic or not removable by surgery (clinical trial No. NCT02538510), displayed therapeutic potential but non-negligible toxicity (299). Nevertheless, it will be necessary to follow the mechanism of vorinostat function and selective targeting of the Shh pathway in OSCC in the future.

## 6 Conclusion

The dysregulation of primary cilia is the biological source of several malignancies, as they are essential for controlling the cell cycle and molecular signalling. However, only few analyses of primary cilia frequency, length and dynamics in individual regions of the oral cavity and their association with tumorigenesis have been performed. Here, we focused on the role of cilia-associated signalling pathways in the pathogenesis of squamous cell carcinoma in the head and neck region. The multifactorial process of HNSCC development is linked to the aberration of many signalling pathways, such as Hh, Wnt, PDGF and EGF. To develop novel targeted treatments for HNSCC, a greater understanding of the complex interaction between cilia and signalling pathways implicated in carcinogenesis is essential. We also paid attention to the possibilities of synthetic inhibitor usage to target signalling pathways dysregulated in HNSCC/OSCC. Recently, several antagonists that inhibit Hh, PDGF, EGF and Wnt signalling have become available for cancer treatment. For solid tumours, these inhibitors are now undergoing clinical trials, and several studies have already revealed their positive effects on squamous cell carcinoma of the head and neck area, which make them a potential target for further rigorous testing.
